# Supplementary material for: WordCommentsAnalyzer: A windows software tool for qualitative research
Source: F1000Res. 2018 Sep 4;7:536. Originally published 2018 May 3. [Version 2] doi: 10.12688/f1000research.14819.2 (PMC6137411; doi:10.12688/f1000research.14819.2)
Supplement: Supplementary file 3 [file f1000research-7-17614-s0002.tgz › b2a79496-4ada-47c4-9682-a99ce867836d.zip!/Eight Iranian Health Journals/Journal of Research in Rehabilitation Sciences _1.docx]

© Journal of Research in Rehabilitation Sciences . This work is licensed under CC BY-NC 4.0 (https://creativecommons.org/licenses/by-nc/4.0/).

Volume & Issue: دوره 13، شماره 4: 1396

Title: تأثیر شتاب خطی و شتاب چرخشی سر در بروز آسیب‌های مغزی در تکواندو

http://jrrs.mui.ac.ir/index.php/jrrs/article/view/2937

مقدمه: مکانیسم‌های آسیب ضربه مغزی به خصوص در ورزش، هنوز به طور کامل شناخته نشده است. هدف از انجام مطالعه حاضر، بررسی شتاب خطی و شتاب چرخشی سر به عنوان عوامل تأثیرگذار در بروز آسیب‌های مغزی در تکواندو و میزان اهمیت آن‌ها در این نوع آسیب‌ها بود. مواد و روش‌ها: در این پژوهش تجربی، 15 تکواندوکار مرد حرفه‌ای شرکت کردند. جهت اندازه‌گیری شتاب خطی و شتاب چرخشی سر، از یک دستگاه متشکل از یک سر و گردن مصنوعی، یک بازوی مکانیکی که نماینده پای تکواندوکار بود و یک مجموعه کامپیوتری جهت ارزیابی، نظارت و ثبت اطلاعات که توسط محقق ساخته شد، استفاده گردید. طراحی و شبیه‌سازی کامپیوتری دستگاه فوق در برنامه‌های SolidWorks، Adams و CATIA انجام شد. نتایج در نرم‌افزار MATLAB به دست آمد و جهت بررسی تأثیر شتاب‌ها در بروز آسیب مغزی از آزمون t استفاده گردید (05/0 > P). یافته‌ها: شتاب خطی به دست آمده در مقایسه با آستانه آسیب سر به طور معنی‌داری کمتر بود (001/0 = P). همچنین، شتاب چرخشی حاصل شده در مقایسه با آستانه برش سطحی مغز (001/0 = P)، تکان مغزی (001/0 = P) و پارگی سیاهرگ ارتباطی (013/0 = P) به طور معنی‌داری بیشتر گزارش شد. نتیجه‎گیری: عامل اصلی آسیب‌های سر در تکواندوکاران، شتاب چرخشی می‌باشد که با توجه به مقادیر به دست آمده در تحقیق حاضر، در صورت عدم استفاده از کلاه می‌تواند منجر به آسیب‌های مغزی جبران‌ناپذیری شود؛ در حالی که شتاب خطی نقش چندانی در تولید این آسیب‌ها ندارد.

Title: مقایسه کینماتیکی مانور فریب بین پای برتر و غیر برتر و رابطه آن با آسیب لیگامان متقاطع قدامی در هندبالیست‌های زن حرفه‌ای

http://jrrs.mui.ac.ir/index.php/jrrs/article/view/2960

مقدمه: تغییر جهت بدن در حرکات سریع ورزشی، از منظر بروز آسیب مورد توجه قرار گرفته است و ساز و کارهای آسیب لیگامان متقاطع قدامی (Anterior cruciate ligament یا ACL) در این زمینه اهمیت ویژه‌ای دارد. تحقیق حاضر به مقایسه کینماتیکی مانور فریب بین پای برتر و غیر برتر و رابطه آن با آسیب ACL پرداخت. مواد و روش‌ها: در این مطالعه، 18 زن هندبالیست حرفه‌ای به‌ صورت هدفمند و در دسترس انتخاب شدند. پس از فرایند نشانه‌گذاری، هر آزمودنی مسافت 3 متر تعیین شده را با سرعت دوید و پس از رسیدن به محل علامت زده شده بر روی زمین، حرکت فریب به سمت پای برتر را نشان داد و به سمت دیگر میله تغییر جهت داد و از آن عبور کرد. سپس مانور را در جهت دیگر و با پای غیر برتر انجام داد. داده‌های کینماتیکی با نرخ نمونه‌برداری 200 هرتز و فیلتر پایین‌گذر 8 هرتز Butterworth، در نرم‌ا‌فزار Cortex پردازش شد. متغیرهای مورد نظر مفصل زانو (فلکشن/ اکستنشن و واروس/ والگوس) نیز با استفاده از آزمون‌های Shapiro-Wilk، Paired t، Levene و ANCOVA در نرم‌افزار SPSS مورد تجزیه و تحلیل قرار گرفت. 05/0 > P به عنوان سطح معنی‌داری در نظر گرفته شد. یافته‌ها: تفاوت معنی‌داری در تغییرات فلکشن (346/0 = P) و اکستنشن (312/0 = P) مفصل زانوی پای برتر و غیر برتر وجود نداشت، اما اختلاف معنی‌د‌اری در تغییرات والگوس (001/0 = P) و واروس (001/0 = P) زانوی پای برتر و غیر برتر مشاهده شد. نتیجه‎گیری: نتایج به دست آمده منجر به شناخت هرچه بهتر خطر شاخص‌های کینماتیکی در وضعیت‌های خطرزا می‌شود. در نتیجه، می‌توان نحوه صحیح اجرای تکنیک‌ها را به ورزشکارانی که در معرض وضعیت‌های پرخطر قرار دارند، آموزش داد. همچنین، بهتر است هنگام اجرای این تکنیک‌ها، از وسایل حمایتی مانند بریس، کنزیوتیپ و زانوبند استفاده گردد.

Title: تغییرات صبحی- عصری تعادل پویا تحت تکلیف دوگانه شناختی در زنان سالمند با عادت صبحگاهی

http://jrrs.mui.ac.ir/index.php/jrrs/article/view/2974

مقدمه: تعییرات ایجاد شده ناشی از افزایش سن در ریتم شبانه‌روزی بدن و عادات زمانی در سالمندان، می‌تواند باعث ایجاد تفاوت در اجرای فعالیت‌های حرکتی و شناختی و همچنين، ترجيحات ساعت روز براي انجام فعاليت‌هاي بدني يا ذهني این افراد نسبت به جوانان شود. توانایی حفظ تعادل در افراد سالمند نیاز به توجه دارد و فعالیت خودکاری نیست و می‌تواند تحت تأثیر این تغییرات قرار گیرد و احتمال زمین خوردن را افزایش دهد. هدف از انجام تحقیق حاضر، بررسی تغییرات صبحی- عصری تعادل پویا در زنان سالمند با عادت صبحگاهی تحت تکلیف دوگانه شناختی بود. مواد و روش‌ها: 12 زن سالمند با دامنه سنی 80-65 سال به صورت هدفمند انتخاب شدند و با قرار گرفتن در سه گروه به صورت تصادفی، جهت اجرای تست زمان برخاستن ‌و‌ رفتن تحت تکلیف دوگانه شناختی و منفرد، در سه روز متفاوت و در دو نوبت صبح و عصر به آزمایشگاه بیومکانیک ورزشی دانشگاه شهید باهنر کرمان مراجعه نمودند. داده‌ها با استفاده از آزمون Two-way repeated measures ANOVA در نرم‌افزار SPSS مورد تجزیه و تحلیل قرار گرفت. یافته‌ها: زمان روز تأثیر معنی‌داری بر عملکرد تعادلی سالمندان هم‌زمان با اجرای تکلیف شناختی (711/0 = P) و بدون اجرای تکلیف شناختی (221/0 = P) نداشت، اما بدون در نظر گرفتن زمان روز، اجرای تکلیف شناختی بر عملکرد تعادلی تأثیرگذار بود (001/0 = P). نتیجه‎گیری: عملکرد تعادلی زنان سالمند تحت تکلیف شناختی و منفرد در عصر نسبت به صبح دچار تغییر نمی‌شود، اما به طور کلی، اجرای تکلیف شناختی هم‌زمان با تست تعادل، تأثیر منفی بر عملکرد تعادلی سالمندان می‌گذارد.

Title: تأثیر تغییر کیفوز سینه‌ای بر طول بازوی گشتاور بار خارجی هنگام برداشتن بار با دو تکنیک اسکات و استوپ در مردان

http://jrrs.mui.ac.ir/index.php/jrrs/article/view/2896

مقدمه: طول بازوی گشتاور بار خارجی، یکی از عوامل تأثیرگذار بر میزان نیروی وارد شده بر ستون فقرات می‌باشد و یکی از تغییراتی است که صرف ‌نظر از نوع تکنیک برداشتن بار، می‌تواند در کاهش خطر ابتلا به کمردرد تأثیرگذار باشد. تغییر در میزان کیفوز سینه‌ای هنگام برداشتن بار، یکی از عوامل مؤثر بر طول بازوی گشتاور بار خارجی در نظر گرفته می‌شود. مواد و روش‌ها: در تحقیق حاضر از 12 آزمودنی مرد درخواست شد تا با استفاده از دو تکنیک اسکات و استوپ در حالی‌ که وضعیت کیفوز سینه‌ای خود را در شروع حرکت فاز اکستنشن افزایش، کاهش و یا بدون تغییر قرار می‌دهند، جعبه‌ای به جرم 10 و 20 درصد جرم بدن خود را از روی زمین بردارند. جهت محاسبه فاصله عرضی بین جعبه و مهره پنجم کمری، از سیستم آنالیز حرکتی استفاده گردید. یافته‌ها: یافته‌های به دست آمده حاکی از تأثیر معنی‌دار نوع تکنیک (001/0 > P)، ارایه دستورالعمل تغییر در کیفوز سینه‌ای (039/0 = P) و جرم جعبه (001/0 = P) بر طول بازوی گشتاور بار خارجی بود. روابط معنی‌داری بین تکنیک و دستورالعمل (012/0 = P)، تکنیک و جرم (026/0 = P)، دستورالعمل و جرم (029/0 = P) و هر سه عامل تکنیک، دستورالعمل و جرم (006/0 = P) وجود داشت. نتیجه‎گیری: طول بازوی گشتاور خارجی با نوع تکنیک، جرم بار خارجی، الگوی حرکتی و پوسچر ستون فقرات در ارتباط است. به‌ کارگیری تکنیک اسکات، افزایش طول بازوی گشتاور بار خارجی را به همراه دارد و افزایش جرم جعبه به‌ طور کلی و افزایش کیفوز سینه‌ای در تکنیک اسکات، باعث کاهش طول بازوی گشتاور می‌گردد.

Title: تعيين اثربخشی مداخله آموزشي آگاهی واج‌شناختی بر افزایش آگاهی واج‌شناختی دانش‌آموزان دارای کاشت حلزون فارسی زبان پايه اول دبستان‌های شهر اهواز

http://jrrs.mui.ac.ir/index.php/jrrs/article/view/2844

مقدمه: كودكان ناشنوا بخشي از دوره زبان‌آموزی را به دليل فقدان ورودي شنوايي از دست می‌دهند و در بيشتر موارد رشد زبان آن‌ها با دشواری‌هايي روبه‌رو می‌شود. یکی از جنبه‌های زبان، آگاهی واج‌شناختی نام دارد. مهارت‌ واج‌شناختی را می‌توان مقدمه‌ای بر رشد خواندن در آینده دانست و آموزش آگاهی واج‌شناختی، منجر به رشد مهارت‌های رمزگشایی می‌شود. پژوهش حاضر با هدف تعيين اثربخشی مداخله آموزشي آگاهی واج‌شناختی بر افزایش آگاهی واج‌شناختی دانش‌آموزان دارای کاشت حلزون فارسی زبان پايه اول دبستان انجام گرفت. مواد و روش‌ها: 28 کودک دارای کاشت حلزون كه مطابق با معیارهای مطالعه بودند، به صورت تصادفی به دو گروه آزمایش و شاهد (هر گروه 14 نفر) تقسیم شدند و پس از اخذ رضايت‌نامه آگاهانه از والدين آن‌ها، هر دو گروه از نظر ميزان آگاهي واج‌شناختي مورد ارزيابي قرار گرفتند. سپس آموزش آگاهي واج‌شناختي بر روي گروه آزمایش انجام گردید. سپس آموزش آگاهي واج‌شناختي بر روي گروه آزمایش انجام شد و در نهایت، ميزان آگاهي واج‌شناختي دو گروه مجدد بررسی گردید. يافته‌ها: ميانگين نمره مهارت آگاهي واج‌شناختي گروه آزمایش پس از مداخله نسبت به گروه شاهد افزايش داشت (۰۰۱/0 = P). همچنين، نمره ميانگين هر يك از خرده آزمون‌های مهارت آگاهي واج‌شناختي در گروه آزمایش نسبت به گروه شاهد چنين افزايشي را نشان داد (050/0 = P برای تمام خرده آزمون‌ها). در این میان، بیشترین و کمترین تأثیر به ترتیب مربوط به مهارت تقطیع و آگاهی از قافیه بود.نتیجه‌گیری: برنامه آموزشی طراحی شده منجر به بهبود مهارت‌های آگاهی واج‌شناختی در كودكان كاشت حلزون می‌شود. بنابراین، استفاده از این برنامه در مداخلات درمانی توصيه مي‌گردد.

Title: تأثیر و ماندگاری خستگی عضلات اندام تحتانی بر زمان رسیدن به پایداری در پرش فرود تک پای مردان بزرگسال هندبالیست لیگ برتر ایران

http://jrrs.mui.ac.ir/index.php/jrrs/article/view/2884

مقدمه: افزایش زمان رسیدن به پایداری، منجر به بروز آسیب می‌شود. بنابراین، اهمیت خستگی در موضوع آسیب و بررسی ارتباط آن با زمان رسیدن به پایداری، می‌تواند نتایج مفیدی را به همراه داشته باشد. هدف از انجام مطالعه‌ حاضر، بررسی تأثیر و ماندگاری خستگی عضلات اندام تحتانی در مفاصل بر زمان رسیدن به پایداری در حرکت پرش فرود در مردان هندبالیست‌ بود. مواد و روش‌ها: 10 هندبالیست لیگ برتر ایران که در سال 1394 در لیگ حضور داشتند، در این پژوهش شركت كردند. سه تکرار برای پای فرود در نظر گرفته شد و داده‌های نيروهاي عکس‌العمل زمين از لحظه‌ تماس با صفحه نیرو به مدت 20 ثانیه ثبت گردید. خستگی عضلات مفاصل ران، زانو و مچ پا به فاصله ۲۴ ساعت برای هر مفصل مورد سنجش قرار گرفت. سپس آزمودنی‌ها حرکت پرش فرود را در دو نوبت، بلافاصله و پس از 10 دقیقه از پروتکل خستگی انجام دادند. زمان رسیدن به پایداری نیز در دو جهت قدامی- خلفی و داخلی- خارجی محاسبه گردید. آزمون Shapiro-Wilk جهت بررسی نرمال بودن و آزمون‌های Repeated measures ANOVA و Bonferroni برای ارزیابی فرضیه‌های تحقیق مورد استفاده قرار گرفت. یافته‌ها: تفاوت معنی‌داری بین زمان رسیدن به پایداری در عضلات اینورتور و اورتور مفصل مچ پا در راستای قدامی- خلفی در شرایط مختلف (قبل از خستگی، بلافاصله بعد از خستگی و ده دقیقه پس از خستگی) وجود داشت (001/0 > P)، اما در راستای داخلی- خارجی نتایج معنی‌داری یافت نشد (650/0 = P). همچنین، اختلاف معنی‌داری بین زمان رسیدن به پایداری در عضلات فلکسور و اکستنسور مفصل زانو در راستای قدامی- خلفی و در شرایط مختلف مشاهده گردید (003/0 = P)، اما این یافته‌ها در راستای داخلی- خارجی مفصل زانو معنی‌دار نبود (228/0 = P). بین زمان رسیدن به پایداری در عضلات ابداکتور و اداکتور مفصل ران، در راستای قدامی- خلفی (051/0 = P) و داخلی- خارجی (343/0 = P) تفاوت معنی‌داری یافت نشد. نتیجه‌گیری: خستگی عضلات اندام تحتانی، منجر به افزایش زمان رسیدن به پایداری و افزایش نیروی وارد شده بر پا می‌شود و ممکن است باعث آسیب شود. با طراحی و گنجاندن برنامه‌های تمرینی استقامتی ویژه، شاید بتوان زمان رسیدن به پایداری در حرکات پرش فرود را به حداقل رساند و در نتیجه، خطر بروز آسیب را کاهش داد.

Title: بررسی قابلیت پاسخگویی نسخه فارسی پرسش‌نامه Oxford به درمان فیزیوتراپی در بیماران ایرانی مبتلا به استئوآرتریت زانو

http://jrrs.mui.ac.ir/index.php/jrrs/article/view/2973

مقدمه: هدف اصلی فیزیوتراپی در بیماران مبتلا به استئوآرتریت زانو، کاهش درد، بهبود عملکرد، افزایش سطح کیفیت زندگی و توانایی‌های فردی است. بنابراین، درمانگران به یک ابزار سنجش پیامد جهت ارزیابی این تغییرات پس از درمان نیاز دارند. ابزارهای اندازه‌گیری پیامد بیمارمحور، باید قابلیت تکرارپذیری، اعتبار و پاسخگویی قابل قبولی داشته باشند. مقیاس پیامد Oxford Knee Score (OKS) از جمله ابزارهایی است که در ارزیابی بیماران مبتلا به استئوآرتریت زانو استفاده می‌شود و تاکنون مطالعه‌ای در جهت بررسی قابلیت پاسخگویی این ابزار به درمان فیزیوتراپی در این گروه از بیماران انجام نگرفته است. هدف از انجام پژوهش حاضر، بررسی قابلیت پاسخگویی مقیاس OKS و اندازه‌گیری حداقل نمره تغییر بالینی مهم در فیزیوتراپی بیماران مبتلا به استئوآرتریت زانو بود. مواد و روش‌ها: 146 بیمار زن و مرد مبتلا به استئوآرتریت زانو که به تشخیص پزشک متخصص به فیزیوتراپی ارجاع شده بودند، به صورت ساده و غیر احتمالی مورد بررسی قرار گرفتند. آن‌ها به مدت 4 هفته به صورت یک روز در میان (به مدت حداقل 10 جلسه) تحت درمان فیزیوتراپی قرار گرفتند و پرسش‌نامه OKS را قبل و پس از درمان تکمیل نمودند. همچنین، در جلسه آخر پرسش‌نامه لیکرت هفت آیتمی گذشته‌نگر را تکمیل کردند. یافته‌ها: سطح زیر منحنی (78/0) و ضریب همبستگی گاما (59/0)، بالا بودن قابلیت پاسخگویی پرسش‌نامه OKS را نشان داد. حساسیت و ویژگی این مقیاس به ترتیب 50/0 و 91/0 به دست آمد و نمره برش مطلوب آن نیز 5/6 گزارش گردید. نتیجه‌گیری: OKS ابزاری با قابلیت پاسخگویی و حساسیت و ویژگی بالا به درمان فیزیوتراپی در بیماران مبتلا به استئوآرتریت زانو می‌باشد. همچنین، از حداقل نمره تغییر بالینی مهم این ابزار می‌توان جهت افتراق بیماران بهبود یافته از بهبود نیافته و تصمیم‌گیری جهت ترخیص و یا ادامه درمان استفاده نمود.

Title: تأثیر چاقی مرکزی بر آستانه شنوایی زنان و مردان 25 تا 40 ساله

http://jrrs.mui.ac.ir/index.php/jrrs/article/view/2872

مقدمه: دستگاه شنوایی، دستگاه بسیار حساسی می‌باشد که نقشی اساسی را در زندگی انسان ایفا می‌کند. مرض چاقی عبارت است از انباشته شدن مضاعف چربی بدن که می‌تواند از راه‌های گوناگون، اثرات زیانباری بر سلامتی دستگاه‌های حسی و حرکتی همچون بینایی، حس عمقی و... داشته باشد. از جمله دستگاه‌های مهمی که چاقی تأثیر سویی بر آن می‌گذارد، شنوایی است. هدف از انجام پژوهش حاضر، بررسی تأثیر چاقی مرکزی بر آستانه‌های شنوایی بود. مواد و روش‌ها: این مطالعه مقطعی- تحلیلی، بر روی 60 نفر در محدوده سنی 25 تا 40 سال (30 زن و 30 مرد) انجام شد. پس از اندازه‌گیری قد، وزن، دور کمر (Waist circumference یا WC) و محاسبه شاخص توده بدنی (Body mass index یا BMI)، این افراد برای ورود به تحقیق انتخاب شدند. برای نمونه‌ها به ترتیب تاریخچه‌گیری، اتوسکوپی، تیمپانومتری و ادیومتری انجام شد. سپس میانگین آستانه شنوایی سه فرکانس 250، 500 و 1000 هرتز به عنوان آستانه شنوایی فرکانس پایین و میانگین آستانه شنوایی سه فرکانس 2000، 4000 و 8000 هرتز نیز به عنوان آستانه شنوایی فرکانس بالا در نظر گرفته شد. در نهایت، میانگین آستانه‌های شنوایی فرکانس‌های پایین و بالا برای نمونه‌ها (با در نظر گرفتن سن و جنسیت) مورد تجزیه و تحلیل قرار گرفت. یافته‌ها: با مقایسه آستانه شنوایی زنان و مردان، می‌توان به وجود اختلاف معنی‌دار در زنان پی برد (001/0 ≥ P). یافته‌ها نشان دهنده تأثیر چاقی مرکزی بر توانایی شنیدن زنان در فرکانس‌های بالا بود، اما در مردان، چاقی مرکزی بر آستانه شنوایی فرکانس‌های پایین و بالای هیچ‌کدام از گوش‌ها تأثیری نداشت (050/0 < P). نتیجه‎گیری: به نظر می‌رسد که چاقی مرکزی در مردان، بر آستانه شنوایی فرکانس‌های پایین و بالای هیچ‌کدام از گوش‌ها تأثیری ندارد و در مقابل در زنان، تأثیر مشخصی را می‌توان در آستانه شنوایی فرکانس بالا مشاهده نمود.
